# Factors affecting psychological distress or quality of life, and association between psychological distress and quality of life in Korean infertile women

**DOI:** 10.1192/j.eurpsy.2025.2435

**Published:** 2025-08-26

**Authors:** H. Suh, S. Kim

**Affiliations:** 1 Psychiatry, CHA University, School of Medicine; 2 Obstetrics and Gynecology, Fertility Center of CHA Gangnam Medical Center, CHA University School of Medicine, Seoul, Korea, Republic Of

## Abstract

**Introduction:**

Infertile patients are more likely to experience psychiatric illnesses than fertile patients, therefore it is important to have early intervention for psychiatric illnesses in infertile patients.

**Objectives:**

This study aimed to determine the clinical factors affecting psychological distress and quality of life (QoL), and investigate the association between psychological distress and QoL in infertile women.

**Methods:**

This was a prospective cohort study of 100 infertile women who voluntarily agreed to participate at their first visit from November 2018 to May 2019. Psychological distress and QoL were evaluated using the 2 questionnaires (SCREENIVF and FertiQoL) specifically designed for infertility.

**Results:**

The prevalence screening positive for anxiety and depression risk were 42% and 29%, respectively. The number of miscarriage and *in vitro* fertilization (IVF) treatments were significantly associated with helplessness risk factor. Furthermore, women with 2 or more IVF treatments had lower emotional, and mind/body QoL domain scores than women with less than 2 IVF treatments. Regarding the association between the levels of psychological distress and QoL in infertile women, the largest association was observed between helplessness risk factor and mind/body QoL domain (r=-0.795, *p* < 0.001). Patients with more risk factors for psychological distress had the worse emotional, and mind/body QoL domain scores.

**Conclusions:**

The levels of psychological distress were significantly associated with QoL in infertile women. These psychologically vulnerable infertile women may receive psychological evaluations and interventions from various angles with conventional treatments for infertility.

**Disclosure of Interest:**

None Declared

